# Insecticidal activity of the essential oil from fruits and seeds of *Schinus terebinthifolia *Raddi against African malaria vectors

**DOI:** 10.1186/1756-3305-4-129

**Published:** 2011-07-05

**Authors:** Eliningaya J Kweka, Mramba Nyindo, Franklin Mosha, Ary G Silva

**Affiliations:** 1 Tropical Pesticides Research Institute, Division of Livestock and Human Disease Vectors Control, Mosquitoes Section, P.O.Box 3024, Arusha, Tanzania; 2 Kilimanjaro Christian Medical College, Tumaini University, P.O.Box 2240, Moshi, Tanzania; 3 Centro Universitário Vila Velha - UVV. Rua Comissário José Dantas de Melo, 21, Boa Vista, Vila Velha, ES, CEP 29.102-770, Brazil; 4 Tommasi Analítica. Avenida Luciano das Neves, 2016, Divino Espírito Santo, Vila Velha, ES, CEP. 29.107-010, Brazil

## Abstract

**Background:**

Alternative insecticides for the control of malaria and filarial vectors are of paramount need as resistance is increasing among classes of insecticides currently in use in the public health sector. In this study, mosquitocidal activity of *Schinus terebinthifolia *essential oil against *Anopheles gambiae *s.s., *An. arabiensis *and *Culex quinquefasciatus *was assessed in laboratory, semi- field and full- field conditions

**Method:**

Twenty third instar larvae of both *Anopheles gambiae *s.s. and *Cx. quinquefasciatus *were exposed to different dosages of plant extract in both laboratory and semi- field environments. Observation of the mortality response was assessed at intervals of 12, 24, 48 and 72 hours. Adult semi- gravid female mosquitoes were exposed to papers treated with *S. terebinthifolia *and compared with WHO standard paper treated with alphacypermethrin (0.05%).

**Results:**

Gas chromatography, coupled to mass spectrometry, identified 15 compounds from *S. terebinthifolia *extracts, the most abundant identified compound was δ-3-carene (55.36%) and the least was γ-elemene (0.41%). The density of the oil was found to be 0.8086 g/ml. The effective dosages in the insectary ranged from 202.15 to 2625.20 ppm and were further evaluated in the semi- field situation. In the laboratory, the mortality of *Cx. quinquefasciatus *ranged from 0.5 to 96.75% while for *An. gambiae *s.s it was from 13.75 to 97.91%. In the semi- field experiments, the mortality rates observed varied for both species with time and concentrations. The LC_50 _and LC_95 _value in the laboratory was similar for both species while in the semi- field they were different for each. In wild, adult mosquitoes, the KT_50 _for *S. terebinthifolia *was 11.29 minutes while for alphacypermethrin was 19.34 minutes. The 24 hour mortality was found to be 100.0% for *S. terebinthifolia *and 75.0% for alphacypermethrin which was statistically significant (*P *< 0.001).

**Conclusion:**

The efficacy shown by essential oils of fruits and seeds of *S. terebinthifolia *has given an opportunity for further investigation of individual components of these plant extracts and to evaluate them in small- scale field trials.

## Background

In Tanzania, it is estimated that 95% of Tanzanians are living in stable malaria transmission areas. About 17-20 million clinical episodes of malaria are reported per year and 80,000 of all deaths are attributed to malaria annually [1,2]. Malaria has been attributed to 40% of outpatients' attendances across health facilities in Tanzania [2]. The recent statistics in Tanzania indicate that, malaria mortality related cases have decreased from 100,000 [1] to 80,000 deaths [2].

For mosquito vector control, effective insecticides and appropriate techniques for application are highly in demand. The reduced susceptibility statuses of malaria and filarial vectors to the recommended insecticides of choice have posed an alarming situation in public health [3-5]. Both vectors and parasites of malaria have shown resistance selection pressure to insecticides and anti- malarial drugs respectively [4,6-10].

Most programmes for malaria vector control rely on insecticide treated nets (ITNs), indoor residual spraying (IRS) and larval control [11-17] and treatments of diagnosed patients with drugs of choice [8,10]. The insecticides mainly authorized for use in malaria control programmes belong to the pyrethroid class, namely permethrin, deltamethrin, alphacypermethrin and lambdacyalothrin [18], organophosphates [19,20], carbamates [19,21], and chlorophenapyr [22]. The organochloride class is currently restricted for public health vector control use, though it was effective against malaria and other disease vectors [23]. The rest of the insecticide classes have been effective when in combination rather than when applied singly [24]. The use of these synthetic pesticides have been found to have side- effects in non-targeted organisms [25].

Plant phytochemicals have more specific effects and could be usefully integrated with other control measures to design comprehensive, appropriate and effective management protocols with less collateral harm to the environment and non- target species [26]. The poor socio-economic status among individuals in malaria endemic areas has denied them access to protective tools against malaria vectors such as bed nets, IRS and topical repellents [27,28]. Alternative chemical compounds with multi- effects in different stages of mosquito development are needed to complement the compounds which are currently in use. Since ancient times, plant extracts have been in use due to their medicinal and vector control properties [29,30]. Currently, several plant species have shown to be effective when used against different disease vectors [31,32]. In Tanzania, a number of plant species have demonstrated major activity against malaria vectors in the laboratory [32-38] and in full field evaluations [36,37].

*Schinus terebenthifolia *Raddi (Anacardiaceae), commonly known as Brazilian pepper, is an evergreen tree, native to South America, in particular Brazil, Paraguay and Argentina. The fruits are drupes and are green when they are immature, and become dark pink or red, when mature [39], with one dark brown seed per fruit [40]. The essential oil of the vegetative parts has been shown to have non-steroidal anti-inflammatory activity by inhibiting phospholipase A2 [41], acting by competitive inhibition of this specific enzyme [42] due to one of its components, schinolmasticadienoic acid [43]. Its healing activity was also directly related to triterpenoids present in fruits [44]. This essential oil also showed antimicrobial activity by several substances, such as terebinthone, hydroxymasticadienoic, terebinthifolic, and ursolic acids [45], and antifungal activity was also evidenced [46]. The essential oil of fruits and seeds of *S. terebinthifolia *has recently shown larvicidal activity, in a dose dependent way, against third instar larvae of *Stegomyia aegypti*, the dengue fever vector [26]. To date, there are no reports on the use of *S. terebinthifolia *extracts against *An. gambiae *s.s., *An. arabiensis *and *Cx. quinquefasciatus*. Botanical pesticides are preferred in comparison to synthetic pesticides, as they are eco-friendly and biodegradable [47].

Since *An. gambiae *s.l. is mainly found in sub-Saharan Africa [46,47], and *S. terebinthifolia *is an exclusive neotropical plant species, native of South America [37], their geographical distribution range would never overlap. However, both of these species had been anthropically put in contact after the accidental introduction of *A. gambiae *s.l. in northeastern Brazil in 1929, probably from Senegal [48]. The correct identity of the invader, if *An. gambiae *or *An. arabiensis*, is not well established, unless by a modeling proposal that points to *An. gambiae *[47]. Although the mosquito species is believed to have been eradicated from Brazil by the end of 1940s [49], the control efforts ended the epidemics, but low-level and localized rural transmission still persist [50].

Therefore, it was the aim of this study to investigate the biological control activity of the essential oil of *S. terebinthifolia *Raddi *against An. gambiae *s.s., *An. arabiensis *and *Cx. quinquefasciatus *, as well as to assess and quantify the mosquitocidal effect in both laboratory and semi- field situations. The insecticide susceptibility status of a wild population of *An. gambiae *s.l. was evaluated using alphacypermethrin (0.05%) as standard and *S. terebinthifolia *as a new product.

## Materials and methods

### Essential oil extraction, purification, and density determination

Fruits of the Brazilian pepper tree were collected from specimens occurring in the region of Vitória, Espírito Santo, Brazil, and 5.0 kg of those fruits were separated from any impurities in the Laboratory of Plant Ecology, at the Centro Universitário Vila Velha (UVV), and afterwards they were crushed. From that amount, 1.2 kg of crushed fruits and seeds were divided into 12 samples of 100 g, which were extracted for determination of the mean yield and the mean density. The remnant 3.8 kg were also crushed and extracted at a larger scale to produce essential oil for biological assays.

The extraction was made by hydrodistillation in a Clevenger apparatus with the crushed fruits and seeds, during one hour of the extraction process. After extraction, the essential oil was transferred to a glass vial, and its purification was made by separation of the remnant water by freezing, and the essential oil which was kept in liquid phase, was drained from the vial [48].

Both the mean yield and the mean density were gravimetrically determined. For the mean yield, the essential oil produced by each sample of 100 g crushed fruits and seeds were weighed, and the mean density was determined by weighing 1 mL of liquid, in an acclimatized room at 20°C, on an analytical balance with accuracy of 1.0 mg. After extraction, 12 samples of 100.0 g of crushed fruits and seeds were taken as replicates. All of these procedures were performed in the laboratory of Chemical Sciences, UVV.

### Chromatographic Analysis

The identification of the essential oil components was carried out by high resolution gas chromatography analysis, in the Fine Chemistry Laboratory, at Tommasi Analítica. The injection volume was 2 μL, composed of 1.6 mL of a solution of essential oil (30 mg/ml) and 0.4 mL of a solution of hydrocarbon series of C_7_-C_30_, as internal standard, both in *n*-hexane as solvent.

The gas chromatography coupled with mass spectrometry - GC-MS - system used consisted of a gas chromatograph, Thermo Scientific^® ^Ultra GC coupled to a mass spectrometer, Thermo Scientific^®^. The fused silica capillary column used was a DB-5 J & W Scientific (30 m × 0.25 mm × 0.25 mm). Helium was the carrier gas and the column temperature program was increased by 3°C per minute between 60°-240°C. The mass spectra were obtained at 70 eV at a scan rate of 0.84 scan/sec, at the range m/z 40-500. The retention times of sample components and a mixture of *n*-alkanes from C_7_-C_30_, co-injected into the GC-MS system under the same temperature program were used for the calculation of the Arithmetic Retention Index - AI - and the Kovats Retention Index (KI) [47]. Identification of components was based on several methods: the calculated AI and KI and mass spectra. The AI and KI were compared with the ones in the available literature [49], and the mass spectra, compared with the GC-MS spectral library.

### Essential oil dispersion at different concentration

Dimethyl sulfoxide (DMSO) was used as surfactant in making the solutions as the dispensing medium for the essential oil and exposure of larvae to treatment. The parts per million (ppm) was counted as the weight of the final solution made. Mixing 1 mL of the essential oil with 5 mL of DMSO (d = 1.1004 g/mL) plus the weight of 994 mL of water, that was (0.822 g + 5.502 g + 994 g = 1000.344 g). This final weight of a litre of solution thereafter was converted to ppm which was ((0.822*1000)/1000.344). Therefore for a litre of solution this gave 822 ppm. Stock solution was prepared from the essential oil extracted to make a solution of 2465.20 ppm in three litres of water, which was serially diluted to get the lowest concentrations for experiments. The lowest concentration screened against larvae was 80.86 while highest was 2465.20 ppm.

### Larvae bioassays in the laboratory

A series of bowls (diameter 14 cm, depth 10 cm) was prepared in five replicates for each control, blank and treatment performed, and each replicate received 20 larvae, including a control with distilled water, and a blank control with an aqueous solution of 0.50% of DMSO that was used as dispersing medium for the essential oil and exposure of larvae to treatment. The number of serial dilutions that were made to assay for the dosages that were effective to cause mortality were as described in WHO larvae bioassay protocol [50]. Serial dilutions were made from 80.86 ppm to 2465.20 ppm. The range of doses with a mortality effect were selected and considered for the laboratory bioassays. Mortality recordings were taken at 12, 24, 48, and 72 hours for both treatments and control. The dead and moribund larvae were recorded as dead.

### Larval bioassay in the semi- field

Semi- field environment structures used in this study were similar to those described in previous studies [51]. The semi- field evaluation protocol was adopted from the WHO protocol [50]. Similar dosages used in the laboratory were evaluated in the semi- field. A series of bowls (with diameter of 14 cm and depth 10 cm) was prepared in five replicates for each control, blank and treatment performed, and each replicate received 20 larvae, including a control with distilled water, and a blank control with an aqueous solution of 0.50% of DMSO that was used as dispersing medium for the essential oil and exposure of larvae to treatment. Other procedures were performed similar to the larval bioassays in the laboratory.

### Hatchability effect of the essential oil of *S. terebinthifolia *on eggs of *An. gambiae *s.s. and *Cx. quinquefasciatus*

Hatchability is defined as the proportion of eggs hatched in treatment or control to the number of eggs introduced initially. Overnight laid eggs of *An. gambiae s.s*. and *Cx. quinquefasciatus *were introduced in treatments and controls, and then monitored for 3 consecutive days. Twenty eggs of *An. gambiae *s.s. and one egg raft of *Cx. quinquefasciatus *were submerged in water treated with three selected essential oil concentrations (808.6, 1617.2, 2465.20 ppm) separately and compared with untreated dechlorinated water. First instar larvae were counted and picked- up every morning in both control and treatments for the all observations.

### Adult susceptibility test for wild population of *An. gambiae *s.l

Whatman square size filter paper (Ahlstrom Filter Paper, Grade 222, catalogue number 2228-1416) was impregnated with the dispersion of the essential oil using a micropipette at a concentration of 2021.5 ppm. After impregnation, filter papers were treated and laid on clean table to dry in a dark room for 24 h before experiments. These papers were compared with the WHO standard impregnated paper, alphacypermethrin, 0.05% (Batch No. AL083; Control No. 083, Impregnated, June 2010 and expires June 2011). Adult wild female mosquitoes were aspirated from a cowshed and held for 24 hours for blood fed mosquitoes to become semi- gravid before experiments. The susceptibility test was carried out as described in WHO insecticide susceptibility test protocols [52,53].

### Data Analyses

The percentage mortality observed was corrected by Abbott's formula [54]. Analysis of variance (ANOVA) was performed to calculate the mean mortality and standard error in different hours for each concentration for third instar larvae. Chi-square test was used to calculate the statistical difference between the proportions of eggs of *Cx. quinquefasciatus *and *An. gambiae *s.s. hatched for each dosage used and in mortality and knockdown differences for susceptibility test between *S. teberinthifolia *and alphacypermenthrin.

The mortality proportions of *An. gambiae *s.s. and *Cx. quinquefasciatus *in treatments and controls were compared using the Chi- square test. Statistical analysis of the experimental data was performed using PWAS statistics 18.0 (Version 18.0 for Windows, SPSS Inc., Chicago, IL) and MS EXCEL 2003 to find out the knockdown time for 50% (KT_50_), mean and 95% confidence intervals.

## Results

### Essential oil extraction purification and density determination

After purification 110.37 mL of essential oil were obtained, with a density of 0.8086 ± 0.0010 g mL^-1^, representing 2.73 ± 0.25% yield from fresh fruits.

### Chromatographic Analysis

**Figure 1:**
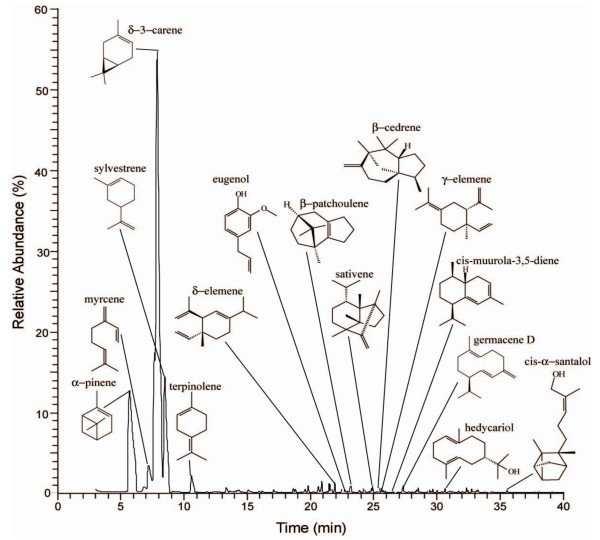
**Chromatographic profile and chemical structure of major constituents of the essential oil from fruits and seeds of *Schinus terebinthifolia***.

The separation of the essential oil components revealed that the chromatographic profile (Figure 1) was composed of 56 substances, mainly mono- and sesquiterpenoids, and among them, only three substances, the monoterpenoids δ-3-carene (55.36%), α-pinene (15.62%), and sylvestrene (10.69%) corresponded to 81.67% of the total composition of the essential oil (Table 1). When 12 more substances are added to the previous ones, a total of 96.11% of the essential oil composition is revealed, including sesquiterpenoids and phenylpropanoids, besides the already cited monoterpenoids. Among those minor compounds that comprised 14.44% of the essential oil composition are germacrene D, β-patchoulene, myrcene, eugenol, terpinolene, sativene, β-cedrene, cis-α-santalol, cis-muurola-3,5-diene, hedycaryol, δ-elemene, γ-elemene. The other 41 compounds occurred in residual traces and comprised only 3.89% of total composition.

**Table 1:** Major constituents of essential oil from fruits of *Schinus terebinthifolia *Raddi, determined by gas chromatography coupled to mass spectrometry and by comparison of their Artihtmetic and Kovats Retention Indices.

Arithmetic Index		Kovats Index			
Calculated	Adams, 2009	Calculated	Adams, 2009	Substance	(%)
1010	1008	1011	1011	δ-3-carene	55.36
933	932	938	939	α-pinene	15.62
1027	1025	1031	1030	sylvestrene	10.69
1479	1480	1481	1481	germacrene D	2.48
1377	1379	1379	1381	β-patchoulene	1.99
988	988	990	990	myrcene	1.89
1355	1356	1357	1359	eugenol	1.71
1087	1086	1089	1088	terpinolene	1.52
1392	1390	1393	1391	sativene	0.93
1418	1419	1419	1420	β-cedrene	0.89
1675	1674	1676	1675	cis-α-santalol	0.77
1447	1448	1449	1450	cis-muurola-3,5-diene	0.76
1546	1546	1548	1548	hedycaryol	0.66
1336	1335	1339	1338	δ-elemene	0.44
1434	1434	1436	1436	γ-elemene	0.41

**Total**					**96.11**

### Larval bioassays in the laboratory

From the range of doses used, the mortality effect observed was from 202.15 ppm to 2625.20 ppm in dechlorinated water. After 72 hours, the mean mortality percentage ranged from 0.5 to 96.75% for *Cx. quinquefasciatus *and 13.75 to 97.91% for *An. gambiae *s.s (Table 2). The interval observations (12, 24, 48, and 72 hours) in each concentration among laboratory experiments had mortality responses that varied with time (Table 2). Overall mortality responses between *An. gambiae *s.s. and *Cx. quinquefasciatus *were statistically significantly different in all doses with more *An. gambiae *s.s. larvae dying (χ ^2 ^= 12.40, *P *≤ 0.0001). The LC_50 _(ppm) in laboratory was similar for both *An. gambiae *and *Cx. quinquefasciatus *1288.71 (1004.20 - 1573.21).

**Table 2:** Overall percentage mortality response of both *Anopheles gambiae *ss and *Culex quinquefasciatus *exposed in different concentrations (ppm) of *S.terebinthifolia *in both laboratory and semi field environments for 72 hours

Study site	Mosquito species	Larval mortality (% mean mortality ± SE)				
		202.15 ppm	404.30 ppm	808.60 ppm	1617.20 ppm	2625.20 ppm
Laboratory	*An. gambiae ss *	13.75 ± 1.20	20.5 ± 1.61	92.91 ± 0.95	93.13 ± 0.96	97.91 ± 0.27
	*Cx. quinquefasciatus*	0.5 ± 0.06	1.25 ± 0.15	74.37 ± 2.24	82.50 ± 1.89	96.75 ± 0.40
Chi-square test		χ ^2 ^= 11.34,*P *< 0.001	χ ^2 ^= 17.18, *P *< 0.001	χ ^2 ^= 11.24, *P *< 0.001	χ ^2 ^= 4.33, *P *= 0.047	χ ^2 ^= 0.005, *P = *0.862
Semi field	*An. gambiae ss *	44.13 ± 0.97	51.96 ± 1.08	62.61 ± 1.01	81.03 ± 1.00	90.64 ± 0.79
	*Cx. quinquefasciatus*	30.78 ± 0.81	38.42 ± 0.96	50.88 ± 1.05	76.37 ± 1.21	89.54 ± 0.89
Chi-square test		χ^2 ^= 3.25,*P *= 0.589	χ^2 ^= 3.17, *P *= 0.062	χ ^2 ^= 2.34,*P *= 0.172	χ ^2 ^= 0.40, *P *= 0.420	χ ^2 ^= 0.001, *P *= 0.817

*Values are mean of five replicates in each dosage

*SE: standard error

### Larvae bioassay in the semi- field

The mortality trends observed in the laboratory assays were similar in semi field experiments for both *Cx. quinquefasciatus *and *An. gambiae s.s*. (Table 2). The interval observations (12, 24, 48, and 72 hours) in each concentration among semi- field experiments had mortality responses that varied with time (Table 3). Mortality of *An. gambiae s.s*. larvae was significantly more than that of *Cx. quinquefasciatus *(χ ^2 ^= 19.27, *P *≤ 0.0001). In semi- field experiments, the overall 72 hour mortality response of *Cx. quinquefasciatus *and *An. gambiae s.s*. in two lower doses (202.15, 404.3) was observed to be higher than observed in laboratory for the same species. In the semi- field, LC_50 _(ppm) for *An. gambiae s.s*. was 847.37 [726.24 - 968.50] while for *Cx. quinquefasciatus *it was 829.88 [719.97 - 939.79].

**Table 3:** Larval mean percentage mortality of *An.gambiae ss *and *Cx. quinquefasciatus *in a concentration gradient of the *S. terebinthifolia *fruits extracts in 12, 24, 48 and 72 hours bioassay

Experiments	Mosquito species	Concentration (ppm)	Larval mortality % (mean ± SE)			
			12 hours	24 hours	48 hours	72 hours
Laboratory	*An. gambiae ss*	Control	0.00 ± 0.00	0.00 ± 0.00	0.00 ± 0.00	0.00 ± 0.00
		Control blank	0.00 ± 0.00	0.00 ± 0.00	4.00 ± 0.28	4.00 ± 0.28
		202.15	0.00 ± 0.00	12.00 ± 0.60	21.50 ± 0.70	29.00 ± 0.20
		404.30	0.00 ± 0.00	13.50 ± 0.70	23.50 ± 0.70	36.50 ± 0.30
		808.60	71.70 ± 1.00	100.00 ± 0.00	100.00 ± 0.00	100.00 ± 0.00
		1617.20	72.50 ± 1.83	100.00 ± 0.00	100.00 ± 0.00	100.00 ± 0.00
		2625.20	91.70 ± 0.17	100.00 ± 0.00	100.00 ± 0.00	100.00 ± 0.00
	*Cx.*	Control	0.00 ± 0.00	0.00 ± 0.00	0.00 ± 0.00	0.00 ± 0.00
	*quinquefasciatus*	Control blank	0.00 ± 0.00	0.00 ± 0.00	3.00 ± 3.00	3.00 ± 3.00
		202.15	0.00 ± 0.00	3.00 ± 0.40	5.00 ± 1.00	5.50 ± 0.90
		404.30	0.00 ± 0.00	0.00 ± 0.00	2.00 ± 0.01	100.00 ± 0.00
		808.60	27.50 ± 0.50	70.50 ± 0.10	99.50 ± 0.10	100.00 ± 0.00
		1617.20	42.50 ± 3.10	87.50 ± 1.30	100.00 ± 0.00	100.00 ± 0.00
		2625.20	87.50 ± 0.10	99.50 ± 0.10	100.00 ± 0.00	100.00 ± 0.00
						

Semi field	*An. gambiae ss*	Control	0.00 ± 0.00	0.00 ± 0.00	0.00 ± 0.00	0.00 ± 0.00
		Control blank	0.00 ± 0.00	0.00 ± 0.00	2.00 ± 2.00	2.00 ± 2.00
		202.15	3.80 ± 0.24	30.30 ± 0.42	64.00 ± 0.33	81.00 ± 0.42
		404.30	13.50 ± 0.13	26.00 ± 0.33	73.50 ± 0.37	95.00 ± 0.52
		808.60	26.50 ± 0.27	37.50 ± 0.34	87.00 ± 0.27	100.00 ± 0.00
		1617.20	27.00 ± 0.29	97.70 ± 0.08	100.00 ± 0.00	100.00 ± 0.00
		2625.20	39.40 ± 0.14	99.20 ± 0.16	100.00 ± 0.00	100.00 ± 0.00
	*Cx.*	Control	0.00 ± 0.00	0.00 ± 0.00	0.00 ± 0.00	0.00 ± 0.00
	*quinquefasciatus*	Control blank	0.00 ± 0.00	0.00 ± 0.00	0.00 ± 0.00	0.00 ± 0.00
		202.15	2.80 ± 0.22	19.00 ± 0.36	34.50 ± 0.38	70.00 ± 0.42
		404.30	5.70 ± 0.19	21.00 ± 0.25	44.00 ± 0.47	84.50 ± 0.31
		808.60	11.80 ± 0.18	34.50 ± 0.31	58.00 ± 0.31	100.00 ± 0.00
		1617.20	11.00 ± 0.25	97.70 ± 0.11	97.80 ± 0.06	100.00 ± 0.00
		2625.20	31.90 ± 0.22	100.00 ± 0.00	100.00 ± 0.00	100.00 ± 0.00

*Values are mean of five replicates in each dosage

*SE: Standard error

### Hatchability effect of *S. terebinthifolia *on eggs of *An. gambiae *s.s. and *Cx. quinquefasciatus*

Hatchability rate was significantly concentration dependent; hatchability rates were 31%, 12.5%, 10% for *An. gambiae s.s*. and *Cx. quinquefasciatus *94%, 49% and 34% in 808.6; 1617.2; 2625.20 ppm respectively (Table 4). The overall proportions hatched between control and treatments were found to be statistically significant (χ ^2 ^= 28.98, *P *< 0.001) with less eggs hatching in treatments. The first instars hatched were found dead at the base of the experiments bowls. After 72 hours, no further hatchability of the remained eggs was observed. The hatching rate was considered as the effect of the essential oil concentration due to 100% hatchability rate in control bowls.

**Table 4:** Hatchability hindrance activity of *S*

Concentration (in ppm)	Mosquito species	Percentage Ovicidal ± SE	Chi-square tests
808.60	*An. gambiae ss *	31.00 ± 1.77	χ ^2 ^= 82.01, *P *< 0.001
	*Cx. quinquefasciatus*	94.00 ± 5.37	

1617.20	*An. gambiae ss *	12.50 ± 1.43	χ ^2 ^= 30.29, *P *< 0.001
	*Cx. quinquefasciatus*	49.50 ± 5.66	

**2625.20**	*An. gambiae ss *	10.30 ± 1.77	χ ^2 ^= 14.94, *P *< 0.001
	*Cx. quinquefasciatus*	34.00 ± 5.83	

*Values are mean of five replicates for each species in each dosage

*SE: standard error

### Adult susceptibility test for wild population of *An. gambiae *s.l

The one hour mosquito knockdown effect comparison between WHO standard impregnated paper alphacypermethrin (0.05%) and the essential oil (1617.20 pmm) impregnated in Whatman filter paper was found to be statistically different (χ ^2 ^= 18.00, *P *< 0.001) with a greater knockdown effect on *S. terebinthifolia*. Alphacypermethrin had KT_50 _(95% Confidence Intervals) value of 19.34 (14.21 - 24. 48) while for the essential oil it was found to be 11.29 (10.10 - 12.48). The overall 24 hour mortality was found to be 100.0% in the essential oil and 85.0% in alphacypermethrin impregnated papers which was statistically significant (χ ^2 ^= 14.13, *P *< 0.001).

## Discussion

The specific gravity of the extracted essential oil was highly significantly lower than the specific gravity previously reported as 0.9097 ± 0.02 g.mL^-1 ^[55], and even that of a previous sample of fruits and seeds processed in this same laboratory, that showed a density of 0.8622 g mL^-1 ^[26]. This was probably because of changes in the quantity and quality of the oil constituents caused by different environmental factors related to soil, sun exposure, amount of water and other external factors. The major compounds found here in the essential oil were the same found by Silva *et al*.,[26] for fruits and seeds of *S terebinthifolia*, with slightest differences concerning their percentage composition [26].

One of the first studies with Brazilian pepper essential oil reported that its main constituents were α-pinene (12.94%), β-pinene (5.02%), α-phellandrene (13.04%), δ-3-carene (29.22%) and β-phellandrene (18.08%) [56]. Another study shows that the major components of this essential oil were δ-3-carene (30.37%), limonene (17.44%), α-phellandrene (12.60%), α-pinene (12.59%), myrcene (5.82%), and *o*-cymene (3.46%)[57]. In another study the main constituents were sabinene, α-pinene, caryophyllene and germacrene D [58]

These qualitative and quantitative variations in this essential oil composition in a same plant species may be related to several different factors. Sometimes it may happen as a consequence of intra-clonal variation in genetic improvement of exclusively vegetative propagated cultivars of rose-scented geranium, *Pelargonium *sp., indicating that even somatic genetics may affect the essential oil biosynthesis and production [59]. In other situations, those differences may be due to environmental factors that may involve geographical origin, or as a consequence of stress produced by abiotic factors such as wind, and air humidity and salinity [60]. The method of cultivation may also affect variations in essential oil composition [61], but even the extraction process must not be disregarded [62].

In this case, the experimental extraction process may be rebutted as a cause for this variation, since the three major compounds were the same that were formerly found, in near the same concentrations, after extraction and chromatographic analysis under exactly in the same conditions [26]. Also, although sourced from the same geographical region, the fruits and seeds were collected at the end of fruiting season. So, those slightest quantitative differences in the major compounds, and the qualitative and quantitative differences on minor compounds [26] could potentially be a consequence of seasonality [63].

The screening of products from this essential oil for ovicidal, larvicidal and adulticidal against mosquitoes might lead to innovative compounds for effective use in the abatement and residual application practice against mosquitoes. The widespread use of synthetic organic insecticides for disease vector control has resulted in an increase of resistance selection pressure in the major disease vector species [3]. This has necessitated the search and development of environmentally safe, biodegradable, low-cost, and indigenous methods for vector control, which can be used without risk of harm to individuals and communities [36,37,54,64]. The control of mosquito-borne diseases can be achieved either by killing, preventing mosquitoes from biting human beings, or by effective usefulness in the abatement and residual application practices in the environment. *S. terebinthifolia *essential oil had found to have no effect on non-targeted organisms as also shown by previous studies [26].

In this study, the laboratory and semi- field findings showed highest efficacy in mortality at lower dosages for the *An. gambiae s.s*. and *Cx. quinquefasciatus *larvae (Table 2 and Table 3). In ovicidal assessment of *An. gambiae s.s*. eggs; hatchability was found to be reduced as the concentration increased, larvae hatched in treated experiments were found dead. In the laboratory, mortality was dosage dependent, while in the semi- field, where other variables such as wind and sunlight where present, the lower dosages had impact on larval mortality which was greater than the effect observed within laboratory experiments (Table 2 and 3). The higher mortality in the lower dosages in the semi- field might be attributed to the degradation of compounds due to exposure to sunlight hence inducing more toxicity. The exposure of plant extracts to sunlight causes molecules to degrade to secondary metabolites which are thought to be the causes of this higher mortalities in semi- field experiments with lower LC_50_[65].

In assessing the adulticidal effect of *S. terebinthifolia *essential oil, using semi- gravid females from a wild population of *An. gambiae s.l*, the time required to knock down 50% of the *An. gambiae *s.l was found to be 6.70 minutes while for the standard WHO recommended paper (alphacypermethrin, 0.05%) it was found to be 27.71 minutes. The KT_50 _attained by this essential oil was more effective than in previous studies conducted using *Ocimum suave *plant extracts [37].

In lower Moshi rice irrigation schemes mosquito populations have been reported to have reduced pyrethroid mortalities due to resistance selection pressure in public health- used pyrethroids [5,24]. In different parts of Africa, pyrethroid insecticides resistance has been observed in adult mosquitoes [3-5] and larvae [66], but the efficacy shown in larvae and adult mortality have proved the ability of the *S. terebinthifolia *essential oil to be formulated and incorporated in control strategies in the community for both larvae and adults. The efficacy shown by *S. terebinthifolia *for knockdown time and 100% mortality after 24 hours to adult mosquitoes from wild resistant population warrants further investigation of these compounds for IRS small scale whether singly or in blends.

This essential oil may be of great value in complementing other compounds which are losing efficacy [67]. From other studies, some phytochemicals have acted as general toxicants against adult as well as larval stages of mosquitoes, while others interfere with growth and development (growth inhibitors) or with reproduction (chemosterilent) or produce olfactory stimuli acting as repellent or attractant [68]. From the last decade, more emphasis has been addressed in larval control for malaria and filarial vector elimination [11-13,16] and control impact has been observed in other areas [11,12]. This is because larval habitat treatment is more localized in time and space resulting in effective control. In tropical countries, plants are known to possess larvicidal, ovicidal and adulticidal activities [34,36-38,69].

The results of our present study in ovicidal, larvicidal and adulticidal properties of the essential oil of fruits and seeds of *S. terebinthifolia *have created the necessity of investigating in detail the ovicidal, larvicidal and adulticidal activities of each chemical compound in this plant extract. In previous studies with *S. terebinthifolia*, Silva *et al. *[26] found high mortality in *Stegomyia aegypti *larvae but no chemical structure was clearly related to these activities shown by fruit extracts.

Further detailed study on the isolated active ingredients responsible for the mosquitocidal activity from these plant fruits may pave the way for the development of an environmentally safe botanical insecticide for the control of mosquitoes at different stages of their life cycle.

## Conclusion

*S. terebinthifolia *offers potential findings against *An. gambiae s.s*., *An. arabiensis *and *Cx. quinquefasciatus *in its marked ovicidal, larvicidal and adulticidal effects. Results of the effect on non- target organisms have revealed that this plant essential oil is safe to certain non- target organisms present in the mosquito habitat. However, further studies of the active ingredients involved and their mode of action are needed to recommend *S. terebinthifolia *essential oil before it can be endorsed as an anti- mosquito product that can be used to combat and protect from mosquitoes and integrated in vector control programmes. Further experiments on sub- lethal dosage exposure of mosquito larvae are ongoing to ascertain the possibility of forming resistant mosquitoes.

## Competing interests

The authors declare that they have no competing interests.

## Authors' contributions

EJK and AGS conceived and designed the study, as well as carrying out the statistical data analyses. AGS carried out the extraction, purification, physical-chemical, and chemical analysis of essential oil, and microbiological bioassays. EJK wrote the manuscript. EJK, AGS, MN and FM revised the manuscript. All authors read and approved this final version.
